# Polarity gene alterations in pure invasive micropapillary carcinomas of the breast

**DOI:** 10.1186/bcr3653

**Published:** 2014-05-08

**Authors:** Nadège Gruel, Vanessa Benhamo, Jaydutt Bhalshankar, Tatiana Popova, Paul Fréneaux, Laurent Arnould, Odette Mariani, Marc-Henri Stern, Virginie Raynal, Xavier Sastre-Garau, Roman Rouzier, Olivier Delattre, Anne Vincent-Salomon

**Affiliations:** 1 INSERM U830, Institut Curie, 26 rue d’Ulm, 75248 Paris Cédex 05, France; 2 Department of Translational Research, Institut Curie, 26 rue d’Ulm, 75248 Paris Cédex 05, France; 3 Department of Tumor Biology, Institut Curie, 26 rue d’Ulm, 75248 Paris Cédex 05, France; 4 Department of Pathology, Centre Georges François Leclerc, and CRB Ferdinand Cabanne, 1 rue Professeur Marion BP 77 980, 21079 Dijon Cédex, France; 5 Department of Surgery, Institut Curie, 26 rue d’Ulm, 75248 Paris Cédex 05, France

## Abstract

**Introduction:**

Pure invasive micropapillary carcinoma (IMPC) is a special type of breast carcinoma characterised by clusters of cells presenting polarity abnormalities. The biological alterations underlying this pattern remain unknown.

**Methods:**

Pangenomic analysis (*n* = 39), *TP53* (*n* = 43) and *PIK3CA* (*n* = 41) sequencing in a series of IMPCs were performed. A subset of cases was also analysed with whole-exome sequencing (*n* = 4) and RNA sequencing (*n* = 6). Copy number variation profiles were compared with those of oestrogen receptors and grade-matched invasive ductal carcinomas (IDCs) of no special type.

**Results:**

Unsupervised analysis of genomic data distinguished two IMPC subsets: one (Sawtooth/8/16) exhibited a significant increase in 16p gains (71%), and the other (Firestorm/Amplifier) was characterised by a high frequency of 8q (35%), 17q (20% to 46%) and 20q (23% to 30%) amplifications and 17p loss (74%). *TP53* mutations (10%) were more frequently identified in the amplifier subset, and *PIK3CA* mutations (4%) were detected in both subsets. Compared to IDC, IMPC exhibited specific loss of the 6q16-q22 region (45%), which is associated with downregulation of *FOXO3* and *SEC63* gene expression. *SEC63* and *FOXO3* missense mutations were identified in one case each (2%). Whole-exome sequencing combined with RNA sequencing of IMPC allowed us to identify somatic mutations in genes involved in polarity, *DNAH9* and *FMN2* (8% and 2%, respectively) or ciliogenesis, *BBS12* and *BBS9* (2% each) or genes coding for endoplasmic reticulum protein, *HSP90B1* and *SPTLC3* (2% each) and cytoskeleton, *UBR4* and *PTPN21* (2% each), regardless of the genomic subset. The intracellular biological function of the mutated genes identified by gene ontology analysis suggests a driving role in the clinicopathological characteristics of IMPC.

**Conclusion:**

In our comprehensive molecular analysis of IMPC, we identified numerous genomic alterations without any recurrent fusion genes. Recurrent somatic mutations of genes participating in cellular polarity and shape suggest that they, together with other biological alterations (such as epigenetic modifications and stromal alterations), could contribute to the morphological pattern of IMPC. Though none of the individual abnormalities demonstrated specificity for IMPC, whether their combination in IMPC may have a cumulative effect that drives the abnormal polarity of IMPC needs to be examined further with *in vitro* experiments.

## Introduction

Breast carcinomas encompass numerous morphologies, phenotypes and molecular alterations [1-3]. Recent comprehensive genomic studies have focused on the most common histological (that is, invasive ductal carcinoma of no special type (IDC-NST) and lobular carcinoma) or molecular (that is, luminal, triple-negative or ERBB2) groups [4-7]. In the present study, we provide a comprehensive molecular characterisation of invasive micropapillary carcinoma (IMPC), one of the special types of invasive breast carcinoma. IMPC represents less than 2% of all invasive breast cancers [2]. This entity is characterised by proliferation of carcinomatous cells organised in clusters, separated from the extracellular matrix by an empty clear space with the cellular apical surface polarised towards the outside. This inverted apical pole is clearly visualised by epithelial membrane antigen (EMA) or MUC1 staining [1] and is the hallmark of this entity.

Up to 70% of patients with IMPC have peritumoural lymphovascular invasion (LVI). This LVI rate is higher than the rates usually observed in IDC-NST (20%). Despite the aggressive phenotype, however, IMPC is associated with a prognosis similar to that of IDC-NST and with the same axillary lymph node status [8-11]. Weigelt *et al*. [3] showed that IMPC are part of the transcriptomic luminal spectrum of tumours, albeit that this observation was based on the analysis of only eight cases. Researchers in two previous genomic studies based on small series of 16 and 10 cases, respectively [12,13], identified specific genomic patterns of IMPC, such as 8p losses, 8q gains and 17p and 16q losses in 50% to 100% of cases. These observations were confirmed by a recent study [14]. Another study subsequently demonstrated that the majority of IMPC cases were oestrogen receptor (ER)–positive and associated with a high proliferation rate and could therefore be considered luminal B tumours [13]. Furthermore, Marchio *et al*. demonstrated the existence of three different genomic patterns: “simplex”, with segments of duplication and deletion of entire chromosomes or chromosome arms; “sawtooth”, with numerous low-level regions of gains or losses; and “firestorm”, with numerous amplifications. In addition, compared to ER- and grade-matched IDC-NST, some genomic alterations were observed more predominantly in IMPC, such as amplification on chromosomes 8p, 8q and 17q.

Despite the results of these previous molecular studies, the biological alterations leading to polarity modifications of IMPC cells associated with high rates of axillary lymph node and LVI have still to be elucidated.

The present study, based on comprehensive genomic analysis by *TP53*, *PIK3CA, SEC63* and *FOXO3* Sanger sequencing, SNP6.0 and transcriptomic analyses of a large series of IMPC and based on whole-exome and RNA sequencing of a subset of cases, identified recurrent mutations in genes enriched in cell polarity, ciliogenesis, cell shape and cytoskeleton organisation that may converge to achieve the observed phenotype but no recurrent fusion genes.

## Methods

### Patients and tumours

We retrospectively selected 77 cases of invasive breast cancer (50 pure IMPC and 27 IDC-NST) on the basis of the availability of both paraffin blocks and frozen specimens from the Institut Curie (45 IMPC and 27 IDC-NST) and Centre Georges François Leclerc (5 IMPC) tumour banks. The initial treatment was surgery in all selected cases. These cases were reviewed by three experienced breast pathologists (AVS, PF and LA) and classified according to the World Health Organization criteria [1]. IMPC cases were confirmed on the basis of inside-out MUC1 staining (cancer antigen 15-3 (CA 15-3), monoclonal antibody, clone DF3; AbCys, Paris, France) at the inverted apical pole [15]. IDC-NSTs were selected as being ER-positive and were grade-matched with IMPC cases.

Whole-exome sequencing was performed for four IMPC cases and their normal DNA (peritumoural), and targeted sequencing validation was performed for forty-seven IMPC cases with a MiSeq Gene & Small Genome Sequencer (Illumina, San Diego, CA, USA). Analysis using the Affymetrix Genome-Wide Human SNP 6.0 Array (Affymetrix, Santa Clara, CA, USA) was feasible for 39 IMPC cases, and whole-transcriptome sequencing was performed for 6 IMPC cases. The types of analyses and number of cases are listed in the flowchart in Additional file 1: Figure S1. All experiments were performed in accordance with French Bioethics Law 2004-800 and the French National Institute of Cancer Ethics Charter and with the approval of the Institut Curie Institutional Review Board and the ethics committees of our institution (“Comité de Pilotage of the Groupe Sein”). The patients gave their written informed consent for us to use their tumour specimens for research. The data were analysed anonymously.

#### Immunohistochemical analyses

Sections (4 μm thick) were cut from formalin-fixed, paraffin-embedded tissues derived from whole tissue sections from representative blocks for each case. These sections were cut, dried, deparaffinised and rehydrated according to standard procedures. All sections were subjected to heat-induced antigen retrieval in citrate buffer (pH 6.1). Antibodies against ERα (clone 6 F11, 1:200; Novocastra, Milton Keynes, UK), ERBB2 (clone CB11, 1:1,000; Novocastra) and MUC1 (clone DF3, 1:100; Bio SB, Goleta, CA, USA) were incubated for 1 hour at room temperature. Staining for horseradish peroxidase antimouse and antirabbit immunoglobulin G was detected with the universal VECTASTAIN Elite ABC Kit (Vector Laboratories, Burlingame, CA, USA) with diaminobenzidine (Dako A/S, Glostrup, Denmark) as chromogen. Internal and external controls were included for each antibody. The American Society of Clinical Oncology–defined cutoffs were used to determine whether cases were positive for ER (≥1%) or for ERBB2 (≥30%) by complete and intense membranous staining [16,17]. MUC1 protein localisation (apical or cytoplasm) was also determined.

#### DNA and RNA extraction

DNA extraction and preparation for microarray experiments of tumour DNA were performed by the Institut Curie Biological Resource Center. Prior to DNA isolation, a tissue section of tumour fragments was obtained and then stained with haematoxylin and eosin to evaluate tumour cellularity. All tumours analysed contained more than 70% of tumour cells on the frozen tissue section after manual microdissection of the frozen specimen. DNA was extracted from frozen tumour samples using a standard phenol/chloroform-based procedure. The quality of DNA was assessed on agarose gels. When a smear instead of a band was observed, the sample was discarded. RNA extractions were performed using a standard previously described procedure [18].

#### Affymetrix Genome-Wide Human SNP 6.0 Array profiling and analysis of genomic alterations

Single-nucleotide polymorphism (SNP) mapping assays were performed according to the manufacturer’s protocol (Affymetrix). Briefly, 250 ng of genomic DNA were digested with both *Nsp* and *Sty* restriction enzymes in independent parallel reactions (Genome-Wide Human SNP Nsp/Sty Assay Kit 6.0; Affymetrix), ligated to the adaptors and amplified by PCR using a universal primer. After purification of PCR products with SNP clean magnetic beads (Agencourt Bioscience, Beverly, MA, USA), amplicons were quantified, fragmented, labelled and hybridised to the Affymetrix Genome-Wide Human SNP 6.0 Array. Targets were prepared when 45 μg of amplified DNA were available and when the targets’ size was situated between 250 and 2,000 bp, and then they were hybridised according to the manufacturer’s recommendations. After washing and staining, the arrays were scanned to generate .cel software files for downstream analysis. Normalisation was performed using a genotyping console (GenomeWideSNP_6.hapmap270.na31.r1.a5.ref) provided by Affymetrix (GTC3.0.1). The data discussed in the present article have been deposited in the National Center for Biotechnology Information (NCBI) Gene Expression Omnibus [GEO:GSE37035] [19]. Genomic alterations were evaluated according to the Genome Alteration Print (GAP) methodology [20]. Copy number and allelic content profiles were detected for each tumour based on the overall pattern of alterations, as previously described and validated [20]. The cutoffs for alteration events (gains, losses and amplifications) were adapted according to the inferred ploidy. For near-diploid tumours, the genomic region with inferred copy numbers ≤1 or ≥3 and ≥6 were considered to be regions of loss, gain and amplification, respectively. For near-tetraploid tumours, the copy number cutoffs used to define regions of loss, gain and amplification were two, six and eight, respectively. The minimal regions of amplification covering at least 25 consecutive SNPs with the same copy number status were considered to be recurrent regions when the frequency of alterations was higher than 20%. Tumour profiles were visualised using GAP software (institut Curie)[20]. Partek GS software version 6.5 build 6.10.1020 (Partek, St. Louis, MO, USA) was used to generate hierarchical clustering of the Affymetrix Genome-Wide Human SNP 6.0 Array genomic data in the IMPC group. Ward’s method with Euclidean distance was used to generate this clustering.

#### The Cancer Genome Atlas breast cancer cohort

The mutational statuses of 358 breast cancers annotated as a luminal subtype (PAM50 [21]) were extracted from The Cancer Genome Atlas (TCGA) database [22]. The frequency of mutations was calculated.

#### Quantitative RT-PCR

cDNA was generated using a reverse transcriptase kit (High Capacity cDNA Reverse Transcription Kit, Applied Biosystems, Foster City, CA, USA) from 1 μg of total RNA. Assays-on-Demand for assessing expression level of *SEC63*, *FOXO3* as well as the control *TATA-binding protein* (*TBP*) genes were obtained from Applied Biosystems. Quantitative RT-PCR was carried out in an ABI PRISM 7500 Real-Time Thermal Cycler using TaqMan Master Mix (Applied Biosystems).

#### Classical Sanger sequencing

Classical Sanger sequences were performed for the following genes: *TP53* (exons 4 to 10; *n* = 45 cases), *PIK3CA* (exons 8, 10 and 21; *n* = 39 cases)*, SEC63* (exons 1 to 21; *n* = 43 cases) and *FOXO3 (*exons 2 to 4; *n* = 51 cases). Each PCR was performed on 30 ng of tumour DNA (*TP53* and *PIK3CA*) or cDNA (*SEC63* and *FOXO3*). These genes were PCR-amplified and bidirectionally sequenced using BigDye Terminator chemistry (Applied Biosystems) with an ABI PRISM 3700 DNA Analyzer. The primer sequences are available in Additional file 2: Table S6. The functional impact of change in amino acids was determined on the basis of scoring using the PolyPhen-2 tool [23].

#### Whole-exome sequencing

Whole-exome sequencing was performed using the SureSelect Human All Exon 50 Mb Kit (Agilent Technologies) and the SOLiD™ experimental tracking software V4 system (Applied Biosystems). Coloured space paired-end reads (50 × 35 bp) were mapped against the UCSC Genome Browser hg19 genome (NCBI build 37.1) using bfast + bwa v0.7.0a [24]. Reads with mapping quality <20 and reads which were marked as duplicates by Picard v1.65 were excluded from further analysis.

Putative somatic single-nucleotide variants (SNVs) were subsequently called in exome data using SAMtools mpileup (v0.1.18 (r982:295)) [25] and VarScan v2.2.11 [26]. False-positive SNVs were excluded using the following thresholds: Fisher’s exact test *P* < 0.05, coverage ≥10×, Phred base quality score ≥20, minimum variant allele frequency ≥20% and high-quality reads supporting variants allele ≥4.

For prioritisation, we omitted (1) known variants from the 1000 Genomes Project and dbSNP Coriell Cell Repository ID V137 and (2) selected missense variants with functional impact scores >0.5 and Genomic Evolutionary Rate Profiling (GERP) scores >3.0. We used the SeattleSeq Annotation 137 server (v7.05, June 2012) [27]. A set of potentially deleterious somatic missense SNVs was validated by classical Sanger sequencing. The validated missense variants were screened in an independent series (*n* = 47) by targeted sequencing on the Illumina MiSeq platform from IntegraGen (Evry, France). The Reactome FI Cytoscape Plugin (National Institute of General Medical Sciences, US National Institutes of Health, Bethesda, MD, USA) was used to determine the observed interactions between the mutated genes identified [28]. For whole-exome and MiSeq sequencing, the average targeted bases covered at 25× were 65% and 68%, respectively.

To determine the functional impact of mutations identified in exome sequencing, PolyPhen-2 (v2.2.2, March 2012) and GERP scores were assessed [23,29]. Accumulative scores were defined on the basis of different predictor tools, including MutationTaster, Condel and MutationAssessor. The thresholds for these combined scores were as follows: for PolyPhen-2, 1 = probably damaging with probable impact on protein structure and function and 0 = benign with no impact on protein structure and function; for GERP, <3 = benign, 3 to 5 = possibly damaging and >5 = probably damaging.

#### RNA sequencing

Library preparation and paired-end (2 × 100 bp) RNA sequencing were performed by IntegraGen using a TruSeq RNA Sample Prep Kit and a HiSeq 2000 platform (both from Illumina), respectively. On average, 130 million reads were obtained for each sample. RNA sequencing raw reads were mapped using TopHat v2.0.6 [30] and bowtie v2.0.4 against the UCSC Genome Browser hg19 genome (NCBI build 37.1). SNV and transcriptome quantitative analysis were performed using SAMtools v0.1.8 and Cufflinks v2.0.2 [31], respectively. Expression level of validated mutations was determined using RNA sequencing data. Gene fusion analyses were performed using two known tools TopHat-Fusion v2.0.4 and deFuse v0.6 (for more detail please refer to Additional file 3: Table S8) [32-34]. Fusion’s validation was performed using RT-PCR (primers listed in Additional file 4: Table S9).

#### Gene expression analysis

The DNA microarray used in this study was the GeneChip Human Genome U133 Plus 2.0 Array (Affymetrix), containing 54,613 probe sets. Microarray data were simultaneously normalised using the GC robust multiarray average package 1.2 in the R environment (R Development Core Team). In this article, expression data are used to determine correlations between the genomic status of genes localised within the chromosome 6q region of minimal deletion and their level of expression by comparing the log_2_ expression signal and the DNA copy number signals using Pearson’s correlation test. A correlation between expression levels with respect to DNA copy number was considered significant when the *R*-value was >0.4 and the *P*-value was ≤0.05.

## Results

### IMPC tumours were predominantly pT1N1 ER-positive with high rates of ERBB2 overexpression for T1 tumours

**Figure 1:**
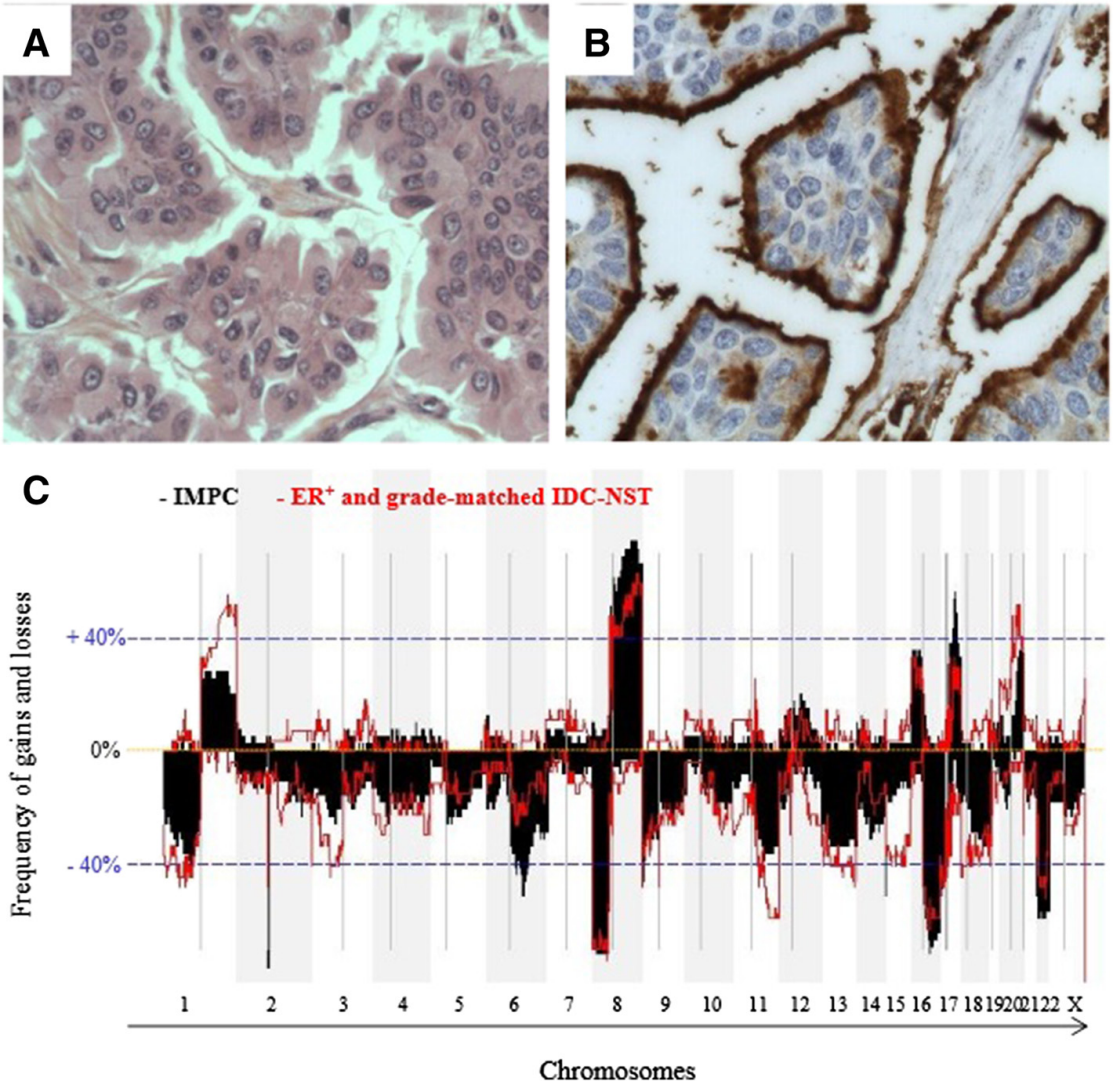
**Phenotypic and genomic characterisation of invasive micropapillary carcinoma. (A)** and **(B)** Histological samples of an invasive micropapillary carcinoma (IMPC) of the breast composed of cell clusters surrounded by empty spaces and displaying an inside-out growth pattern, as highlighted by MUC1 staining. **(C)** Frequency plots of gains and losses are displayed from chromosome 1pter on the left to chromosome Xq on the right. Alternating grey and white bands indicate chromosome boundaries. Dashed blue line represent 40% frequencies, − for losses and + for gains, respectively. ER, Oestrogen receptor; IDC-NST, Invasive ductal carcinoma of no special type.

The aim of this exploratory study was to decipher genomic alterations related to the specific morphology of IMPC tumours, that is, the inverted polarity of cells, organised in clusters separated from the extracellular matrix by a clear space (Figures 1A and 1B). IMPC tumours have previously been identified as luminal carcinomas. The 39 IMPC tumours subsequently analysed by Affymetrix Genome-Wide Human SNP 6.0 Array were therefore compared to 27 ER-positive and grade-matched IDC-NSTs. All clinicopathological data are provided in Additional file 5: Table S1.

On the basis of the PAM50 gene list [35], the transcriptomic analysis of IMPC tumours showed that they were either luminal A (18 of 34 cases) or luminal B (16 of 34 cases) (Additional file 6: Figure S2).

The median ages of the IMPC and IDC-NST patients were 62 and 60 years, respectively, and all patients were treated and followed at the Institut Curie between 1990 and 2010. The majority of IMPC tumours were T1 (72%), grade 2 (51%) and ER-positive (100%), and ERBB2 overexpression was observed in 26% of cases.

### IMPC tumours harboured 17q gain/amplification associated with 6q deletion

The copy number and allelic status of each altered region in the tumour genome were determined in order to assess chromosome number and infer the ploidy of each tumour sample. The number of breakpoints (defined as a change in copy number or allelic status within a chromosome) was evaluated for each tumour. The median numbers of breakpoints in IMPC tumours and in ER-positive and grade-matched IDC-NSTs were almost identical (65 for IMPC tumours and 79 for IDC-NSTs).

Frequencies of gains and losses are shown in Figure 1C. Recurrent amplifications (observed in more than 40% of cases) were identified on chromosomes 8q and 17q, as listed in detail in Table 1. The three most frequent regions of amplification observed in IMPCs were 8q22.1, 8q23.3-q24.23 and 17q22-q23.3, encompassing *RAD54B, CCNE2* and *TP53INP1* (21% *vs* 5% in IDC-NSTs), *MYC* (21% *vs* 14% in IDC-NSTs) and *BCAS3*, *PPM1D*, *TLK2*, *TBX2* and *TANC2* (22% *vs* 7%), respectively. IMPC cases also presented amplifications in the *ERBB2* region (22% *vs* 24% in IDC-NSTs).

**Table 1:** **Frequencies of regions of gains, losses and amplifications in invasive micropapillary carcinomas and in oestrogen receptor–positive and grade-matched invasive ductal carcinomas of no special type**^**a**^

**SNP start**	**SNP end**	**Chr**	**Cytoband**	**IMPCs (%)**	**IDC-NSTs (%)**	**Genes of interest**
**Common regions**						
*Gains*						
36948617	146292734	8	p11.23-q24.3	60	45	
*Losses*						
113565	35436036	8	p23.3-p12	63	63	
46534977	90163275	16	q11.2-q24.3	55^b^	54	
6689	18896297	17	p13.3-p11.2	46	43^b^	
16055171	51219006	22	q11.1-q13.33	52	40	
**Specific regions**						
*Gains*						
197811282	249198692	1	q31.3-q44	23	48	
52579874	67308196	17	q22-q24.3	47	25	
33435161	62648208	20	q13.12-q13.33	17	44	
*Losses*						
101380020	125365648	6	q16.3-q22.31	45^b^	20	
705598	13611533	18	p11.32-p11.21	15	42	
28175177	35313501	18	q12.1-q12.2	24	40	
*Amplifications*						
37097563	38618768	8	p11.23-p11.22	13	26	*FGFR1, PPAPDC1B, WHSC1L1*
94967717	97023919	8	q22.1	21	5	*RAD54B, CCNE2, TP53INP1*
115849871	138121678	8	q23.3-q24.23	21	14	*MYC*
69073647	70229171	11	q13.3-q25	7	22	*CCND1, ORAOV1, FGF19, FGF4, FGF3*
37298761	38225021	17	q12-q21.1	22	24	*STARD3, ERBB2, GRB7*
57159990	62354992	17	q22-q23.3	22	7	*BCAS3, TBX2, TLK2, TANC2, PPM1D*
50695849	52586281	20	q13.2	15	26	*ZNF217, BCAS1*

^a^Chr, Chromosome; IDC-NST: invasive carcinoma of no special type; IMPC, Invasive micropapillary carcinoma; SNP, Single-nucleotide polymorphism. Recurrent gains, losses or amplifications observed in more than 20% of cases after exclusion of genomic variants according to the Database of Genomic Variants (http://dgv.tcag.ca/dgv/app/home). SNP start and SNP end refer to SNP positions corresponding to the boundaries of gains, losses or amplifications. Genomic positions are provided according to human genome 19 (hg19) references in base pairs. ^b^Existence of a homozygous deletion is observed in one case among IMPCs and IDC-NSTs studied. The lower part of the table lists the specific regions of gains, losses and amplifications that were statistically different between IMPCs and IDC-NSTs with *P* < 0.05 by Fisher’s exact test.

**Figure 2:**
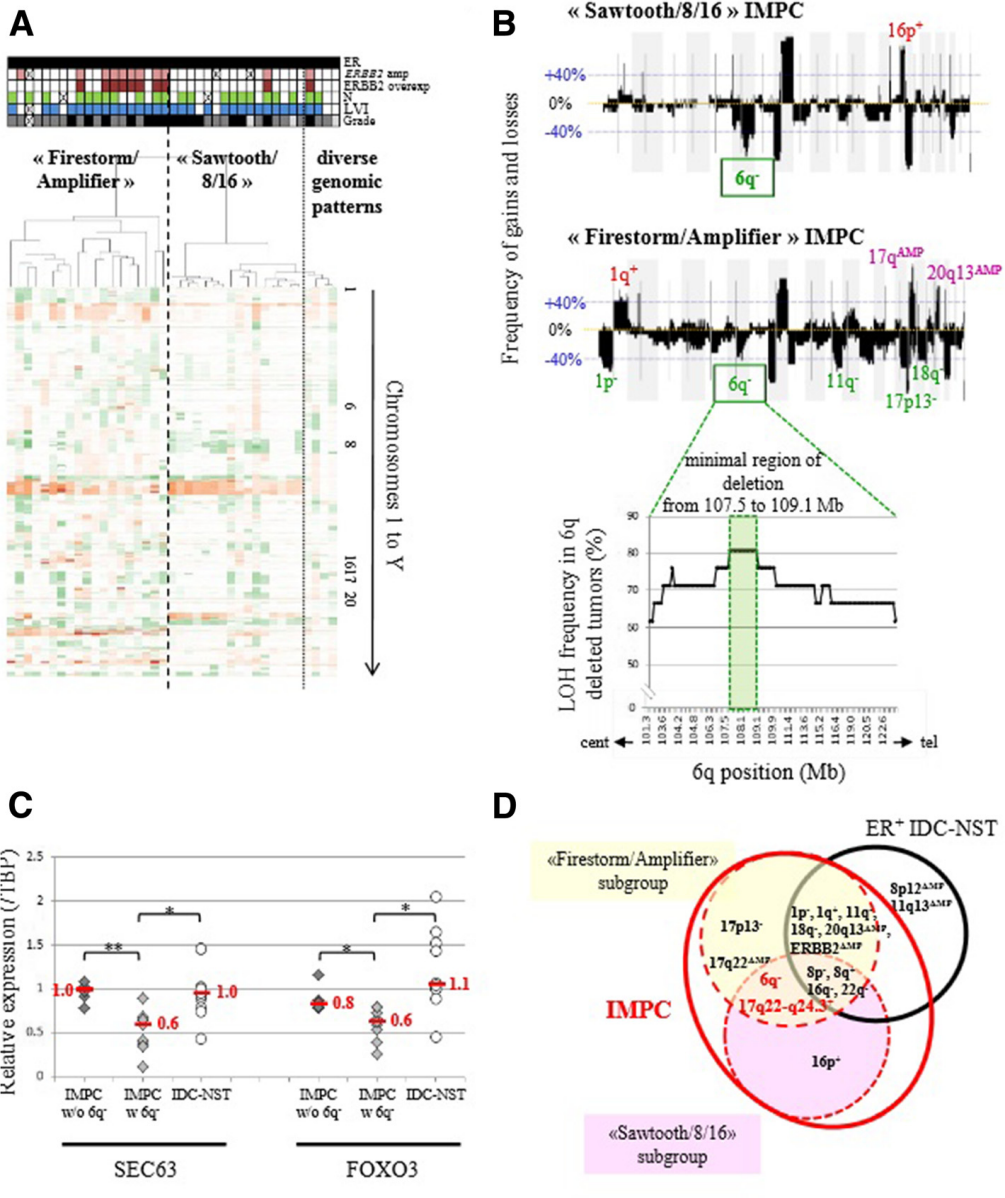
**Identification of two invasive macropapillary carcinoma genomic subgroups. (A)** Unsupervised hierarchical clustering of 39 (IMPC) tumours using Affymetrix Genome-Wide Human SNP 6.0 Array data with Partek software. Each column represents a different tumour, and each row represents single-nucleotide polymorphism (SNP) status (green for losses, red for gains and/or amplifications). Colour coding in lines above the dendrogram: black in top row = Oestrogen receptor–positive (ER+); pink = *ERBB2* amplification; brown = ERBB2 overexpression; green = Lymph node metastasis (N+); blue = Lymphovascular invasion–positive (LVI+); pale grey = grade 1; dark grey = grade 2; black in bottom row = grade 3. **(B)** Frequencies of genome copy number gains and losses plotted as a function of genome location in “Sawtooth/8/16” IMPC (upper panel) and “Firestorm/Amplifier” IMPC (middle panel). Gains and losses are displayed above or below the blue line, respectively, from chromosome 1pter on the left to chromosome Xq on the right. Alternating grey and white bands indicate chromosome boundaries. The regions exhibiting significantly more frequent gains or losses between “Sawtooth/8/16” IMPC and “Firestorm/Amplifier” IMPC are indicated above and below the frequency plots, respectively. Frequency of loss of heterozygosity (LOH) of chromosome 6q assessed on the basis of Affymetrix Genome-Wide Human SNP 6.0 Array profiles observed in the tumours presenting a deletion of the chromosome 6 long arm. The minimal region of deletion is highlighted. cent, Centromere; tel, Telomere. **(C)** Real-time PCR results of relative expression of *SEC63* and *FOXO3* genes in samples of IMPC without (*n* = 6; dark-grey diamond) and with (*n* = 9; light-grey diamond) chromosome 6q deletion and in invasive ductal carcinoma of no special type (IDC-NST) (*n* = 11; circle) (in red: median relative value/Tata Binding Protein). w/o, without; w, with. *P ≤ 0.05, **P ≤ 0.001. **(D)** Summary of the common and distinct genomic alterations between IMPC genomic subgroups and ER+ IDC-NSTs.

As expected, some of the most frequent recurrent changes (observed in at least 40% of cases) were those commonly found in luminal B breast cancers, with gains of 8q being associated with losses of 8p, 16q, 17p and 22q [36]. However, compared to IDC-NSTs, IMPCs specifically harboured more gains of chromosome 17q22-q24.3 (47% *vs* 25%) and more losses of chromosome 6q16.3-q22.31 (45% *vs* 20%) (Table 1). This chromosome 6q loss was associated with loss of heterozygosity (Table 1 and Figure 2B).

### Identification of two different genomic subsets of invasive micropapillary carcinomas

Heterogeneous copy number profiles with a high frequency of recurrent regions of amplification and frequent combinations of recurrent alterations (such as chromosome 8p loss, 8q gain, 16q loss, 17q and/or 20q amplifications) observed in the IMPC group prompted us to search for genomic subsets. By unsupervised clustering analysis of IMPC data derived from the Affymetrix Genome-Wide Human SNP 6.0 Array, we identified two major clusters among IMPCs (Figure 2A). The first cluster was composed of 19 cases (48%) harbouring regions of amplification located on chromosomes 8q, 17q and 20q, with frequencies ranging from 20% to 46%, hereinafter called the “Firestorm/Amplifier” subset [37]. The second cluster was composed of 16 cases (41%) harbouring rare regions of amplification, but whole-arm copy number alterations such as 8p−/8q+/16p−/16q+, hereinafter called the “Sawtooth/8/16” subset [37].

Different gains and losses distinguished “Firestorm/Amplifier” from “Sawtooth/8/16” subsets of IMPCs (detailed in Figure 2B and Additional file 7: Table S2). Differences in genomic alterations between each of these two IMPC subsets and IDC-NSTs were then identified (presented in Additional file 8: Table S3 and Additional file 9: Table S4). In contrast to their genomic differences, the two IMPC subsets harboured identical rates of grade 3 LVI- and ER-positive cases and the same clinical stage distribution (size and N+ number) (Additional file 10: Table S5).

### *SEC63* and *FOXO3* genes, localised in 6q minimal region of deletion observed in both genomic groups of IMPCs, downregulation and mutation

**Figure 3:**
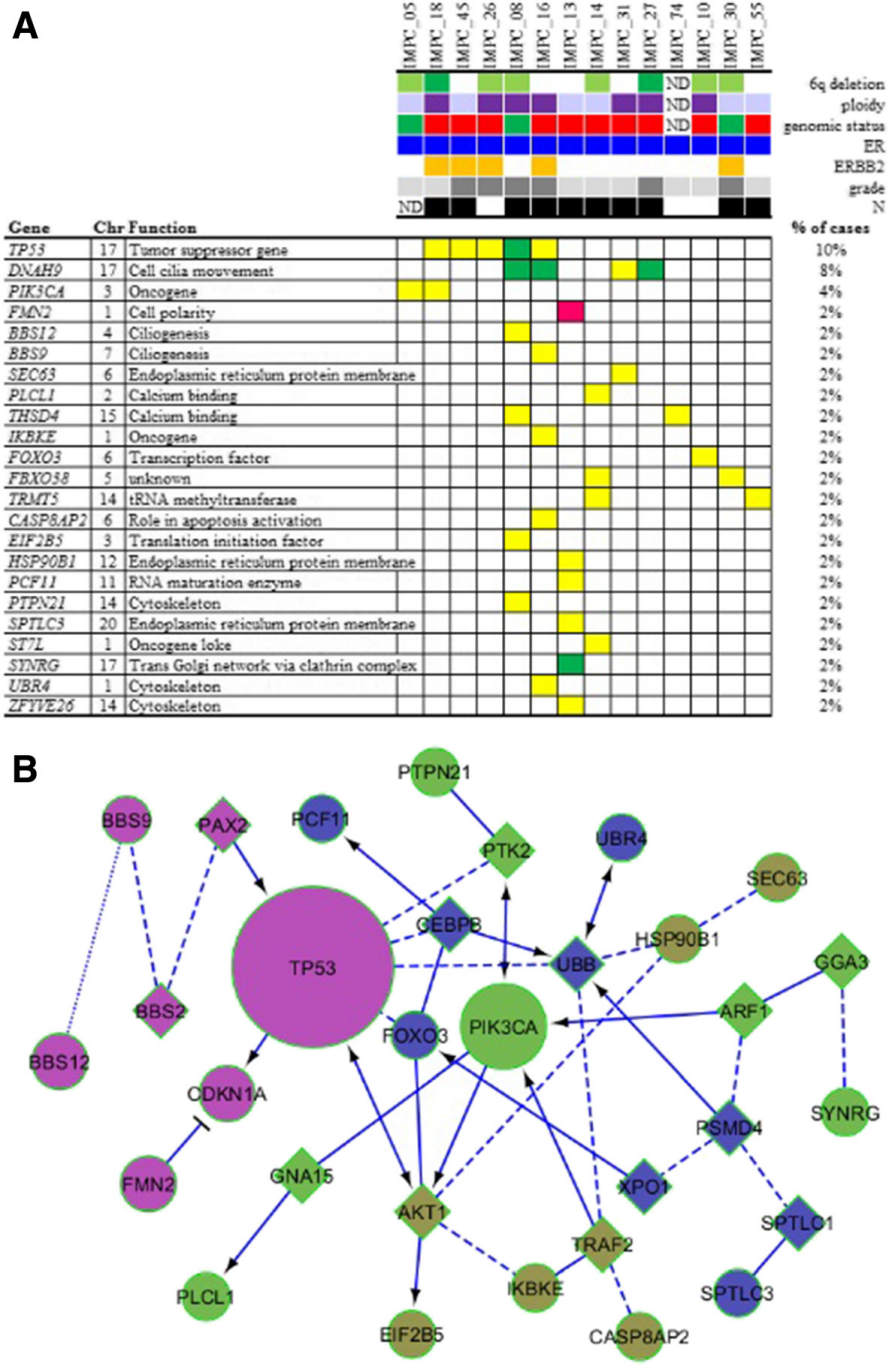
**Mutation landscape in invasive micropapillary carcinoma according to grade, genomic subgroup and phenotype and pathway interactions between the 23 mutated genes. (A)** Distribution of validated somatic mutations among the 50 cases as determined by whole-exome sequencing, targeted MiSeq sequencing and classical Sanger sequencing (*TP53, PIK3CA, SEC63 and FOXO3*). Central heatmap: distribution of significant mutations across sequenced samples with color-coding according to the allelic genomic status determined by Affymetrix Genome-Wide Human SNP 6.0 Array analysis. Yellow = neutral copy number; green = deletion, dark pink = gain. Upper chart color-coding from top to bottom: chromosome 6q allelic status (light green = loss of heterozygosity; dark green = deletion), tumour ploidy (dark purple = tetraploid; light purple = diploid), invasive micropapillary carcinoma (IMPC) genomic group (red = “Firestorm/Amplifier”; green = “Sawtooth/8/16”), oestrogen receptor (ER) status (dark blue = positive), ERBB2 overexpression (orange = positive 3+), grade (light grey = grade 1; intermediate grey = grade 2; dark grey = grade 3), axillary lymph node status (dark = N+). Chr, chromosome; ND, Not determined. Function is defined according to the Gene Ontology database and UCSC Genome Browser. **(B)** Interactions and pathways between the 23 mutated genes in IMPCs deduced from the Reactome algorithm [38]. In this diagram, the functional interaction (FI) network connections are symbolised by arrows for activating/catalysing, solid lines ending in perpendicular line for inhibition, solid lines for FIs extracted from complexes or inputs, dashed lines for predicted FIs and dotted lines for defined FIs.

We delineated a 1.59-Mb minimal region of 6q deletion, observed in 45% of all IMPC cases. This region was observed more frequently in IMPCs than in all IDC-NSTs (Figure 2B) and luminal B IDC-NSTs (Additional file 11: Table S7 and Additional file 12: Figure S3). This deletion was further associated with a loss of heterozygosity specific to IMPCs. This region encompassed nine known genes according to the UCSC Genome Browser (GRCh37/hg19, February 2009). Two of the nine genes, *SEC63* and *FOXO3*, encompassed by the common region of deletion were underexpressed in IMPC cases (*P* ≤ 0.05 by Welch test, fold change ≥1.5, real-time quantitative RT-PCR) (Figure 2C) and were consequently sequenced. Primers used for sequencing are listed in Additional file 7: Table S6. One of the thirty-eight cases (IMPC 10; 2% of cases) presented three *FOXO3* missense mutations (Figure 3 and Table 2). One of forty-one cases, IMPC 31, presented a *SEC63* missense mutation (Figure 3 and Table 2). On the basis of its PolyPhen-2 score of 1, this mutation was considered deleterious.

**Table 2:** Mutations in invasive micropapillary carcinomas

**Gene**	**Cases,** ***n***	**(%)**	**Chr**	**Mutation**	**WH seq**	**MiSeq**	**Sanger seq**	**Effect of the mutation**	**Type of mutation**	**PolyPhen-2 score**	**GERP score**	**RNA-seq expression**
*TP53*	5	(10)	17	GAA>CAA/p.E286Q	X	NA	X	Missense	Transversion	1.00		Yes
				ATG>ACG/p.M246T	X	NA	X	Missense	Transition	1.00		Yes
				GAG>AAG/p.E68K	NA	NA	X	Missense	Transition	0.09		ND
				TAC>TGC/p.Y234C	NA	NA	X	Missense	Transition	0.97		ND
				c.742_743insA	NA	NA	X	Frame shift	Ins/del		5.91	ND
*DNAH9*	4	(8)	17	A>G	X	X	X	Splice intron	Transition		4.05	No coverage
				CGG>CAG/p.R2605Q	X	X	X	Missense	Transition	1.00		No coverage
				ATG>ATA/p.M3430I	NA	X	X	Missense	Transition	0.00		ND
				CAG>TAG/p.Q3082*	NA	X	X	Nonsense	Transition		4.05	ND
*FBXO38*	2	(4)	5	TAT>TGT/p.Y1058C	X	X	X	Missense	Transition	1.00		Yes
				GAA>CAA/p.E20Q	NA	X	X	Missense	Transversion	0.96		ND
*THSD4*	2	(4)	15	GAG>AAG/p.E476K	X	X	X	Missense	Transition	1.00		Yes
				GAG>AAG/p.E652K	NA	X	X	Missense	Transition	0.96		ND
*TRMT5*	2	(4)	14	TTT>CTT/p.F303L	X	X	X	Missense	Transition	0.04		Yes
				GAG>CAG/p.E152Q	NA	X	X	Missense	Transversion	0.03		ND
*PIK3CA*	2	(4)	3	CTC>GTC/p.L540V	NA	NA	X	Missense	Transversion	0.99		ND
				CAG>CCG/p.Q546P	NA	NA	X	Missense	Transversion	1.00		ND
*FOXO3*	1	(2)	6	GCA>ACA/p.A267T	NA	NA	X	Missense	Transition	0.97		ND
				CCT>TCT/p.P292S	NA	NA	X	Missense	Transition	0.34		ND
				TTG>GTG/p.L528V	NA	NA	X	Missense	Transversion	0.03		ND
*BBS12*	1	(2)	4	CGC>TGC/p.R674C	X	X	X	Missense	Transition	1.00		Yes
*BBS9*	1	(2)	7	CCA>TCA/p.P77S	X	X	X	Missense	Transition	1.00		No coverage
*CASP8AP2*	1	(2)	6	GAT>AAT/p.D1420N	X	X	X	Missense	Transition	1.00		No coverage
*EIF2B5*	1	(2)	3	GCA>ACA/p.A406T	X	*	X	Missense	Transition	0.05		Yes
*FMN2*	1	(2)	1	GCT>CCT/p.A659P	X	*	*	Missense	Transversion	0.74		No coverage
*HSP90B1*	1	(2)	12	ACG>ATG/p.T468M	X		X	Missense	Transition	0.98		Yes
*IKBKE*	1	(2)	1	GAC>TAC/p.D571Y	X	*	X	Missense	Transversion	0.68		Yes
*PCF11*	1	(2)	11	AAT>AGT/p.N167S	X	X	X	Missense	Transition	0.02		Yes
*PLCL1*	1	(2)	2	AAG>AAT/p.K279N	X	X	X	Missense	Transversion	0.98		No coverage
*PTPN21*	1	(2)	14	CGA>CTA/p.R864L	X	X	X	Missense	Transversion	1.00		Yes
*SEC63*	1	(2)	6	CGC>TGC/p.R217C	NA	X	X	Missense	Transition	1.00		ND
*SPTLC3*	1	(2)	20	G>A	X	X	X	Splice intron	Transition		5.91	No coverage
*ST7L*	1	(2)	1	GAT>GCT/p.D339A	X	X	X	Missense	Transversion	1.00		Yes
*SYNRG*	1	(2)	17	ATG>ATA/pM970I	X	*	X	Missense	Transition	0.45		Yes
*UBR4*	1	(2)	1	AAC>AAA/p.N3400K	X	X	X	Missense	Transversion	0.98		Yes
*ZFYVE26*	1	(2)	14	CAA>CCA/p.Q1582P	X	X	X	Missense	Transversion	0.00		Yes

^a^%, Percentage of mutated cases out of the sequenced cases; *Assessed but not identified with the technique; Chr, Chromosome; GERP, Genomic Evolutionary Rate Profiling; IMPC, Invasive micropapillary carcinoma; NA, Not assessed with this technique; ND, Not determined (that is, no RNA sequence available for that sample); No coverage, Absence of aligned reads at the corresponding genomic position; WH seq, Whole-exome sequencing; X, Assessed and identified with the technique; Yes, Mutated allele is expressed.

### Invasive micropapillary carcinoma DNA and RNA sequencing analysis

We conducted whole-exome sequencing analyses of four IMPC cases (one “Sawtooth/8/16” and three “Firestorm/Amplifier”), followed by targeted sequencing analyses (MiSeq) of forty-seven IMPC cases. Twenty-nine tumour-specific, nonsynonymous variants were identified, twenty-two of which were validated by classical Sanger sequencing.

Altogether, 36 nonsynonymous variants were identified by classical Sanger, whole-exome and targeted (MiSeq) sequencing. These nonsynonymous variants were located in 23 different genes and observed in 14 of the 47 IMPC samples (30%). One-third of the mutated samples harboured more than one nonsynonymous variant. These nonsynonymous variants corresponded to missense mutations in 89% (32 of 36) of cases and transition in 22 of 36 cases (61%). They were considered deleterious according to PolyPhen-2 score (higher than 0.5) and GERP score (higher than 4) in 26 (72%) of 36 cases (Table 2).

**Figure 4:**
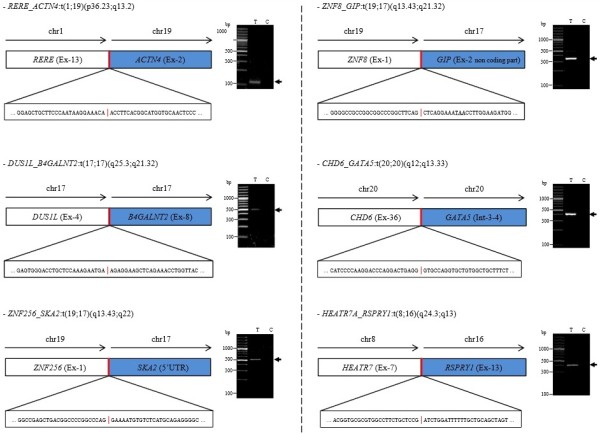
**RNA sequencing identification and validation of the six fusions found in invasive micropapillary carcinoma.** Schematic representations of the fusions between gene 1 and gene 2 are displayed with the precise sequence of the breakpoint region. In the *ZNF8_GIP* fusion scheme, the underlined codon is a stop codon. RT-PCR detection of fusion genes in tumour (T) and constitutional (C) RNAs are shown on the right panel for each fusion. The numbers of split and spanning reads, together with the genomic strands of the fused genes, are given in Additional file 3: Table S8. Primer sequences used for RT-PCR validation are provided in Additional file 4: Table S9. chr, Chromosome; UTR, Untranslated region.

RNA sequencing identified seven putative fusion genes. Six of them were further validated (Figure 4): *RERE-ACTNA4* t(1;19)(p36.23;q13.2), *HEATR7-RSPRY1* t(8;16)(q24.3;q13), *ZNF8-GIP* t(19;17)(q13.43;q21.32), *ZNF256-SKA2* t(19;17)(q13.43;q22), *DUS1L-B4GALNT2* t(17;17)(q25.3;q21.32) and *CHD6-GATA5* t(20;20)(q12;q13.33). All but one of the fusions were private events and observed in one of the thirty-three cases analysed. The *ZNF8-GIP* fusion was observed in two of thirty-three cases. In addition, two IMPC samples demonstrated several fusion genes (two and three fusion genes per sample). These cases were also associated with point mutations.

### Invasive micropapillary carcinomas harboured mutations in genes involved in polarity, ciliogenesis and cell shape

The three most frequently mutated genes in our series of IMPCs were *TP53* (10%), *DNAH9* (8%) and *PIK3CA* (4%) (Figure 3A). Among the cases with only *TP53* mutations, two also had mutations in genes involved in ciliogenesis (*DNAH9*, *BBS12* and *BBS9*) or in cytoskeleton organisation (*UBR4* and *PTPN21*). Among the eight cases without *TP53* or *PIK3CA* mutations, three demonstrated multiple mutations (two to six mutations per case). These multiple mutations are located in genes coding for proteins that play a key role in cell polarity (*FMN2* and *SEC63* (one case (2%) each) or in biological processes necessary for cell polarity or cell shape, such as ciliogenesis (*DNAH9* (two cases (4%), or cytoskeleton (*ZFYVE26* (one case (2%)). Interactions between the 23 mutated genes and existing signalling pathways were investigated with the Reactome algorithm and are shown in Figure 3B. Fifteen (68%) of twenty-two of the validated mutations were found to be expressed at the RNA level, which was strongly correlated with Affymetrix Genome-Wide Human SNP 6.0 Array gene expression levels (*R* = 0.86) found in the RNA sequencing data.

## Discussion

In this study, we show that IMPC (1) were luminal and predominantly pT1N1 with high rates of ERBB2 overexpression, (2) were associated with 17q gain/amplification and 6q deletion, (3) encompassed two different genomic subsets (Firestorm/Amplifier or Sawtooth/8/16), (4) harboured *SEC63* and *FOXO3* gene downregulation and mutation (2% of cases each) and (5) were localised in the 6q minimal region of deletion. We also describe the genomic landscape of pure micropapillary carcinomas by using tools that enabled us to gain insight into the mutations in genes participating in polarity, ciliogenesis, cell shape and cytoskeleton organisation and private translocation at high resolution.

IMPCs presented with complex genomic profiles and numerous gains, losses or high-level amplifications as well as numerous breakpoints, and, in line with their luminal phenotype, demonstrated a genomic profile that shared similarities with that of ER+N+LVI+ IDC-NSTs with regard to 8q gains and 8p, 16q and 22q losses. Some of these alterations have previously been reported to be preferentially associated with luminal B group carcinomas, such as 8q gains, 8p and 16q losses [14,36,39]. However, the high resolution of the present analysis allowed us to identify differences between the two groups, in particular chromosome 17q22-q24.3 gains and 6q16.3-q22.31 losses in IMPCs.

In the present study, we show not only that these 6q losses exist, confirming a previous report of chromosome 6q losses by Marchio *et al.*[13]*,* but also show that they were associated with allelic losses in this region. We also found a correlation between genomic status and transcriptomic expression level of the genes located within this 6q region. Two of these genes, *SEC63* and *FOXO3,* were downregulated. We identified a missense mutation in *SEC63* and three mutations at different positions in *FOXO3* (Table 2). Both the SEC63 and FOXO3 proteins have recently been identified as participating in cell polarity control. *SEC63* encodes for an endoplasmic reticulum protein involved in the early stage of multispanning membrane protein synthesis [40] and has been found to be mutated in polycystic liver and kidney diseases [41]. SEC63 protein also participates in quality control and trafficking of proteins necessary for ciliogenesis, and therefore for epithelial cell polarity [42]. FOXO3 is known to regulate *LKB1* gene transcription, and the LKB1 protein plays a key role in cell planar polarity during cell division [43]. *FOXO3* downregulation has also recently been reported in high-grade serous ovarian carcinoma [44].

The IMPCs we studied had amplification rates of 20%, considered to be high for such early tumours (stage T1). One of the most frequently recurrent regions of amplification observed in IMPCs was located on chromosome 17q22-q23.3. Notably, this region is the hallmark of the cluster 1 of tumours of the METABRIC classification [7,45] and encompasses druggable genes belonging to the kinase family, such as *TLK2* and the phosphatase *PPM1D*. A the second frequently amplified recurrent region was the *ERBB2* region, which was amplified in 22% of cases, a higher rate than that observed in other T1 and small T2 IDCs [46,47].

Two of the most frequent regions of amplification in the IDC-NST control group were located on chromosome 8p and on 11q13.3, which that encompass the *FGFR1* and *PPAPDC1B* genes [48] and the *CCND1* gene, respectively. The combination of the *CCND1* 11q and 8p regions of amplification has previously been reported to be a characteristic of luminal carcinomas [49,50]. The low frequency of amplification of these regions in IMPCs is another genomic difference between luminal IDC-NSTs and IMPCs.

Not surprisingly, *TP53* and *PIK3CA* were among the three most recurrent mutated genes in this series of IMPCs. Although *TP53* mutation rates were similar to those reported in ER-positive breast carcinomas [7], it is noteworthy that *PIK3CA* mutations were observed less frequently in IMPCs than in other ER+ histological subgroups [14,51]. Notably, the number of *PIK3CA* mutations observed in our series of pure IMPCs is much lower than a rate reported recently (four of twenty pure and mixed invasive micropapillary carcinomas) [52].

*DNAH* gene family mutations were recently identified in triple-negative breast carcinomas, albeit at a very low frequency (3%) [53]. In our present study of a special type of breast cancer, we identified recurrent mutations of the *DNAH9* gene (8% of the cases) by whole-exome sequencing analysis, a rate higher than that reported for luminal IDCs in the TCGA analyses (3%). Dynein proteins are necessary for correct apical and apicobasal localisation of the CRUMBS [54] and PAR3 [55] proteins. Recurrent *DNAH9* mutations in IMPC, characterised by a polarity defect, may suggest a causative role of these mutations in the IMPC pattern of growth that remains to be demonstrated *in vitro.*

*FMN2,* a formin-like 2 gene, and the *BBS9* and *BBS12* genes also harboured mutations, although they were not recurrent. These genes are involved in cell polarity, organisation and motility [56] or ciliogenesis [57]. We noted that the *BBS9* and *BBS12* genes were not found to be mutated in the TCGA luminal series. Interestingly, in our particular series, these mutations are always associated with multiple other mutations (from four to five) and located in genes coding for proteins that play a role in cytoskeleton organisation (*HSP90B1, ZFYVE26, UBR4* and *PTPN21*).

These genes have also been identified to be mutated in triple-negative breast carcinomas [4], which raises the hypothesis of their role in tumour cell invasion and motility enhancement in triple-negative tumours or IMPCs. All of these observations have to be confirmed with the sequence of a larger number of IMPC cases at a higher depth. The clinical impact of our results is not yet straightforward. However, better knowledge of polarity disorganisation in breast carcinomas and its role in tumour progression could lead to new therapeutic strategies if confirmed in a larger series. In agreement with the findings of Natrajan *et al.*[14], we observed that IMPCs are not associated with recurrent fusion genes.

## Conclusion

In our comprehensive genomic analysis of IMPCs of the breast, we identified numerous genomic alterations and somatic mutations, but no recurrent fusion gene. We demonstrate that IMPC is associated with a specific genomic profile compared to ER+LVI+N+ IDC-NSTs, consisting of gains of 17q, high rates of 17q22-23.3 and 17q12 *ERBB2* amplifications and losses of 6q heterozygosity. The presence of the *SEC63* and *FOXO3* genes located in this 6q loss of heterozygosity associated with deleterious somatic mutations suggests that they could play a role in the abnormal polarity of IMPC cells. Considered together, the mutation spectrum observed in IMPCs shows that, in addition to *TP53* and *PIK3CA* mutations, some IMPCs harboured mutations in particular in genes involved in ciliogenesis, polarity maintenance and cell shape (often several such mutations per case). However, not all the cases harboured mutations in these biological processes, suggesting that other biological alterations (for example, epigenetic modifications, stromal alterations) could contribute to the morphologically specific pattern of IMPCs, knowing that this hypothesis should be further supported by *in vitro* experiments.

## Abbreviations

dbSNP: National Center for Biotechnology Information database of single-nucleotide polymorphism; EMA: Epithelial membrane antigen; ER: Oestrogen receptor; IDC: Invasive ductal carcinoma; IDC-NST: Invasive ductal carcinoma of no special type; IMPC: Invasive micropapillary carcinoma; LVI: Lymphovascular invasion; METABRIC: Molecular Taxonomy of Breast Cancer International Consortium; N+: Lymph node metastasis.; PR: Progesterone receptor; SNP: Single-nucleotide polymorphism; SNV: Single-nucleotide variant; TBP: Tata-binding protein.

## Competing interests

The authors declare that they have no competing interests.

## Authors’ contributions

NG performed the experiments, interpreted the data and wrote the manuscript. JB performed the bioinformatics analyses, interpreted the data and contributed to the critical revision of the manuscript. VB, TP and MHS performed the SNP6.0 data GAP analysis and contributed to the critical revision of the manuscript. VB and VR performed part of the experiments and contributed to the critical revision of the manuscript. OM prepared the nucleic acids and contributed to the critical revision of the manuscript. PF and LA provided IMPC samples and contributed to the critical revision of the manuscript. RR and XSG participated in the study design and collection of the clinical data and contributed to the critical revision of the manuscript. OD and AVS designed the study, interpreted the data and wrote the manuscript. All authors read and approved the final manuscript.

## Supplementary Material

Additional file 1: Figure S1 Study flow-chart. Click here for file

Additional file 2: Table S6 Primers used for sequencing *SEC63 and FOXO3* genes. Click here for file

Additional file 3 **Two tables. Table S8A**: deFuse post analysis filtering. **Table S8B**: Fusion candidates that were common in TopHat-Fusion and deFuse analysis and had been validated by RT-PCR technique. Click here for file

Additional file 4: Table S9 RT-PCR primer sequences for fusion validation. Click here for file

Additional file 5: Table S1 Clinical and pathological characteristics. Click here for file

Additional file 6: Figure S2 Intrinsic subtype classification. Intrinsic subtype classification was performed using the PAM50 predictor [35] for all invasive micropapillary carcinoma (IMPC) with available transcriptomic analysis. Samples are displayed in columns, and genes are shown in lines. Above the heatmap, the ERBB2 status (in red: overexpression; in white: no overexpression) and the genomic IMPC subgroups (in yellow: Firestorm/Amplifier; in green: Sawtooth/8/16) are indicated. The IMPCs were distributed either in the luminal B group (16 (47%) of 34) or in the luminal A group (18 (53%) 34). Click here for file

Additional file 7: Table S2 Frequencies of common and specific gains and losses in Sawtooth/8/16 and Firestorm/Amplifier invasive micropapillary carcinoma subsets. Click here for file

Additional file 8: Table S3 Frequencies of common and specific regions of gains and losses in Firestorm/Amplifier invasive micropapillary carcinoma subgroup and invasive ductal carcinoma of no special type. Click here for file

Additional file 9: Table S4 Frequencies of common and specific regions of gains and losses in Sawtooth/8/16 invasive micropapillary carcinoma subgroup and invasive ductal carcinoma of no special type. Click here for file

Additional file 10: Table S5 Comparison of frequency plots of invasive micropapillary carcinoma and luminal B invasive ductal carcinoma of no special type. Frequency plots of gains and losses are displayed from chromosome 1pter on the left to chromosome Xq on the right. Alternating grey and white bands indicate chromosome boundaries. Dashed blue line represent 40% frequencies, − for losses and + for gains, respectively. IMPC, Invasive micropapillary carcinomas; Luminal B IDC-NST, Luminal B invasive ductal carcinoma of no special type. Click here for file

Additional file 11: Table S7 Frequencies of common and specific regions of gains and losses in invasive micropapillary carcinoma and luminal B invasive ductal carcinoma of no special type. Click here for file

Additional file 12: Figure S3 Clinical, pathological characteristics, and treatments of patients and tumours in the two genomic subgroups of invasive micropapillary carcinoma. Click here for file
