# Supplementary material for: Polarity gene alterations in pure invasive micropapillary carcinomas of the breast
Source: Breast Cancer Res. 2014 May 8;16(3):R46. doi: 10.1186/bcr3653 (PMC4095699; doi:10.1186/bcr3653)
Supplement: Additional file 1: Figure S1 — Study flow-chart. [file bcr3653-S1.pdf]

50 IMPC tumors

- SNP6.0 (n=39)
- MiSeq (n=47)
- *TP53* classical Sanger sequencing (n=43)*
- *PIK3CA* classical Sanger sequencing (n=41)$
- Whole exome sequencing (n=4)#
- Whole transcriptome (n=6) #

*: 39 out 43 tumors have been analyzed on SNP6.0 arrays too
$: 39 out 41 tumors have been analyzed on SNP6.0 arrays too
#: analyzed on SNP6.0 arrays and MiSeq too

Tumor	SNP6.0 arrays	whole exome sequencing	MiSeq	whole transcriptome sequencing	*TP53* mutation	*PIK3CA* mutation
IMPC_01	X		X		X	X
IMPC_02	X		X		X	X
IMPC_03	X				X	X
IMPC_04	X		X		X	X
IMPC_05	X		X		X	X
IMPC_06	X		X		X	X
IMPC_07	X				X	X
IMPC_08	X	X	X	X	X	X
IMPC_09	X				X	X
IMPC_10	X		X		X	X
IMPC_12	X		X		X	X
IMPC_13	X	X	X	X	X	X
IMPC_14	X	X	X	X	X	X
IMPC_16	X	X	X	X	X	X
IMPC_17	X		X		X	X
IMPC_18	X		X		X	X
IMPC_19	X		X		X	X
IMPC_20	X		X	X	X	X
IMPC_21	X		X	X	X	X
IMPC_26	X		X		X	X
IMPC_27	X		X		X	X
IMPC_29	X		X		X	X
IMPC_30	X		X		X	X
IMPC_31	X		X		X	X
IMPC_32	X		X		X	X
IMPC_33	X		X		X	X
IMPC_34	X		X		X	X
IMPC_36	X		X		X	X
IMPC_38	X		X		X	X
IMPC_39			X		X	
IMPC_41	X		X		X	X
IMPC_42	X		X		X	X
IMPC_43	X		X		X	X
IMPC_44	X		X		X	X
IMPC_45	X		X		X	X
IMPC_47			X		X	
IMPC_49	X		X		X	X
IMPC_50	X		X		X	X
IMPC_55	X		X		X	X
IMPC_57	X		X		X	X
IMPC_59	X		X		X	X
IMPC_60			X		X	X
IMPC_61			X		X	X
IMPC_66			X			
IMPC_69			X			
IMPC_70			X			
IMPC_72			X			
IMPC_74			X			
IMPC_75			X			
IMPC_76			X			

Supplementary Figure 1
