# Supplementary material for: Polarity gene alterations in pure invasive micropapillary carcinomas of the breast
Source: Breast Cancer Res. 2014 May 8;16(3):R46. doi: 10.1186/bcr3653 (PMC4095699; doi:10.1186/bcr3653)
Supplement: Additional file 2: Table S6 — Primers used for sequencing SEC63 and FOXO3 genes. [file bcr3653-S2.pdf]

Supplementary Table 6: Primers used for sequencing *SEC63 and FOXO3* genes.

Name	Size	Sequence
SEC63_Left_1	19	5'-agtgcagagcgtggtcgtg_3'
SEC63_Right_1	21	5'-ggatcaaattcagaagctcca-3'
SEC63_Left_2	22	5'-cccgaaatatggatatgaaacg-3'
SEC63_Right_2	20	5'-tcttctgttgccatcctcct-3'
SEC63_Left_3	22	5'-cgtcacactctactgcacttcc-3'
SEC63_Right_3	22	5'-caaatatgtgcaaacactgtgg-3'
FOXO3_Left_1	20	5'-gtccctgaagggaaggaagc-3'
FOXO3_Right_1	20	5'-ctgtcgcccttatccttgaa-3'
FOXO3_Left_2	20	5'-acaaacggctcactctgtcc-3'
FOXO3_Right_2	20	5'-tagagctccgctgcatgagt-3'
FOXO3_Left_3	20	5'-atatggcaggcaccatgaat-3'
FOXO3_Right_3	20	5'-agtttgagggtctgctttgc-3'
