# Supplementary material for: Polarity gene alterations in pure invasive micropapillary carcinomas of the breast
Source: Breast Cancer Res. 2014 May 8;16(3):R46. doi: 10.1186/bcr3653 (PMC4095699; doi:10.1186/bcr3653)
Supplement: Additional file 3 — Two tables. Table S8A: deFuse post analysis filtering. Table S8B: Fusion candidates that were common in TopHat-Fusion and deFuse analysis and had been validated by RT-PCR technique. [file bcr3653-S3.pdf]

Supplementary Table 8A: deFuse post analysis filtering.

deFuse Filters	Threshold
: number of split reads supporting the prediction	≥ 2
: number of spanning reads supporting the fusion	≥ 5
: *p*-value, lower values are evidence the prediction is a false positive	> 0.1
: *p*-value, lower values are evidence the prediction is a false positive	
: *p*-value, lower values are evidence the prediction is a false positive	
: maximum percent identity of fusion sequence alignments to EST islands	< 0.3
: maximum percent identity of fusion sequence alignments to EST	
: maximum percent identity of fusion sequence alignments to cDNA	< 0.1
: maximum percent identity of fusion sequence alignments to genome	
: number of spanning reads that map to more than one genomic location	< 1
: fusion involving adjacent potentially resulting from co-transcription rather than genome rearrangement (Readthrough)	NO
: fusion between adjacent genes	
: fusion likely the product of alternative splicing between adjacent genes	
: probability produced by classification using adaboost	≥ 0.75
: fusion combines genes in a way that preserves a reading frame	YES

Supplementary Table 8B: Fusion candidates that were common in TopHat-Fusion and deFuse analysis and had been validated by RT-PCR technique.

Gene name 1	Gene name 2	Strand	Spanning read count	Split read count	Gene location 1	Gene location 2	Genomic break pos 1	Genomic break pos 2	Library name	Probability
DUS1L	*B4GALNT2*	-/+	69	49	coding	coding	80021344	47241450	IMPC_13	0.99
ZNF256	*SKA2*	-/-	32	29	5'utr	coding	57232703	58458813	IMPC_13	0.85
RERE	*ACTN4*	-/+	10	11	coding	coding	8482787	39191240	IMPC_14	0.98
ZNF8	*GIP*	+/-	50	92	coding	5'utr	58790513	47044615	IMPC_13	0.99
HEATR7A	*RSPRY1*	+/+	36	71	coding	coding	145235426	57265079	IMPC_21	0.95
CHD6	*GATA5*	+/-	211	437	coding	intron	40034129	61044084	IMPC_16	0.76
