# Supplementary material for: Polarity gene alterations in pure invasive micropapillary carcinomas of the breast
Source: Breast Cancer Res. 2014 May 8;16(3):R46. doi: 10.1186/bcr3653 (PMC4095699; doi:10.1186/bcr3653)
Supplement: Additional file 4: Table S9 — RT-PCR primer sequences for fusion validation. [file bcr3653-S4.pdf]

Primers name	Primers sequence
Fus_RERE_ACTN4_Left	5’-TCAGGCAGTACGGGAAGAAC-3’
Fus_RERE_ACTN4_Right	5’-GATCTGTGTGCCTGCCTTC-3’
Fus_DUS1L_B4GALNT2_Left	5’-CCAACTACCGGAAGGAGAAC-3’
Fus_DUS1L_B4GALNT2_Right	5’-AGAACGTATTTGGTGGTGAC-3’
Fus_ZNF256_SKA2_Left	5’-TCATAAATCTGGCATGTGAAA-3’
Fus_ZNF256_SKA2_Right	5’-TGGACTGATTACTTGGTAGAAA-3’
Fus_ZNF8_GIP_Left	5’-CCATGGCAATACTGTAGTCA-3’
Fus_ZNF8_GIP_Right	5’-GGAGTGCCTCTCTGGTCT-3’
Fus_CHD6_GATA5_Left	5’-ATACTGCAGGAGACAAAGGA-3’
Fus_CHD6_GATA5_Right	5’-AGTCTGCTTGACTGGCTAAG-3’
Fus_HEATR7_RSPRY1_Left	5’-AGACAGTTCATCAGCAAGGT-3’
Fus_HEATR7_RSPRY1_Right	5’-TCTCGGATACTGACTTGCTTC-3’

Supplementary Table 9 : RT PCR primers sequences for fusion’s validation
