# Supplementary material for: Polarity gene alterations in pure invasive micropapillary carcinomas of the breast
Source: Breast Cancer Res. 2014 May 8;16(3):R46. doi: 10.1186/bcr3653 (PMC4095699; doi:10.1186/bcr3653)
Supplement: Additional file 5: Table S1 — Clinical and pathological characteristics. [file bcr3653-S5.pdf]

Supplementary Table 1: Clinical and pathological characteristics.

	IMPC [n (%)]	IDC-NST [n (%)]	*p*-val
Number of patients	39	27	
Median FU, yrs [range]	6.3 [0.6 ; 10.7]	4.0 [0.9 ; 13]	
Median age, yrs [range]	62 [34 ; 84]	60 [38 ; 85]	
< 50	8 (21)	7 (26)	*ns*
> 50	31 (79)	20 (74)	*ns*
Tumor size, cm			
< 2	28 (72)	6 (22)	7.4×10^{-5}
2 to 5	9 (23)	17 (63)	1.1×10^{-3}
> 5	2 (5)	4 (15)	*ns*
Grade			
I	3 (8)	5 (19)	*ns*
II	20 (51)	14 (52)	*ns*
III	15 (38)	8 (30)	*ns*
Not specified	1 (3)	0 (0)	
ER			
Positive	39 (100)	27 (100)	*ns*
Negative	0 (0)	0 (0)	
ERBB2			
Positive	10 (26)	7 (26)	*ns*
Negative	26 (66)	16 (70)	
Not specified	3 (8)	1 (4)	

Legends: Median FU, yrs: median follow-up in years; ER: estrogen receptor; IMPC: invasive micropapillary carcinoma; IDC-NST: invasive carcinomas of no special type.
