# Supplementary figures and images for: Polarity gene alterations in pure invasive micropapillary carcinomas of the breast
Source: Breast Cancer Res. 2014 May 8;16(3):R46. doi: 10.1186/bcr3653 (PMC4095699; doi:10.1186/bcr3653)

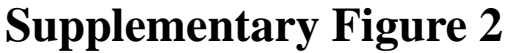

Supplement: Additional file 6: Figure S2 — Intrinsic subtype classification. Intrinsic subtype classification was performed using the PAM50 predictor [35] for all invasive micropapillary carcinoma (IMPC) with available transcriptomic analysis. Samples are displayed in columns, and genes are shown in lines. Above the heatmap, the ERBB2 status (in red: overexpression; in white: no overexpression) and the genomic IMPC subgroups (in yellow: Firestorm/Amplifier; in green: Sawtooth/8/16) are indicated. The IMPCs were distributed either in the luminal B group (16 (47%) of 34) or in the luminal A group (18 (53%) 34). [file bcr3653-S6.pdf]

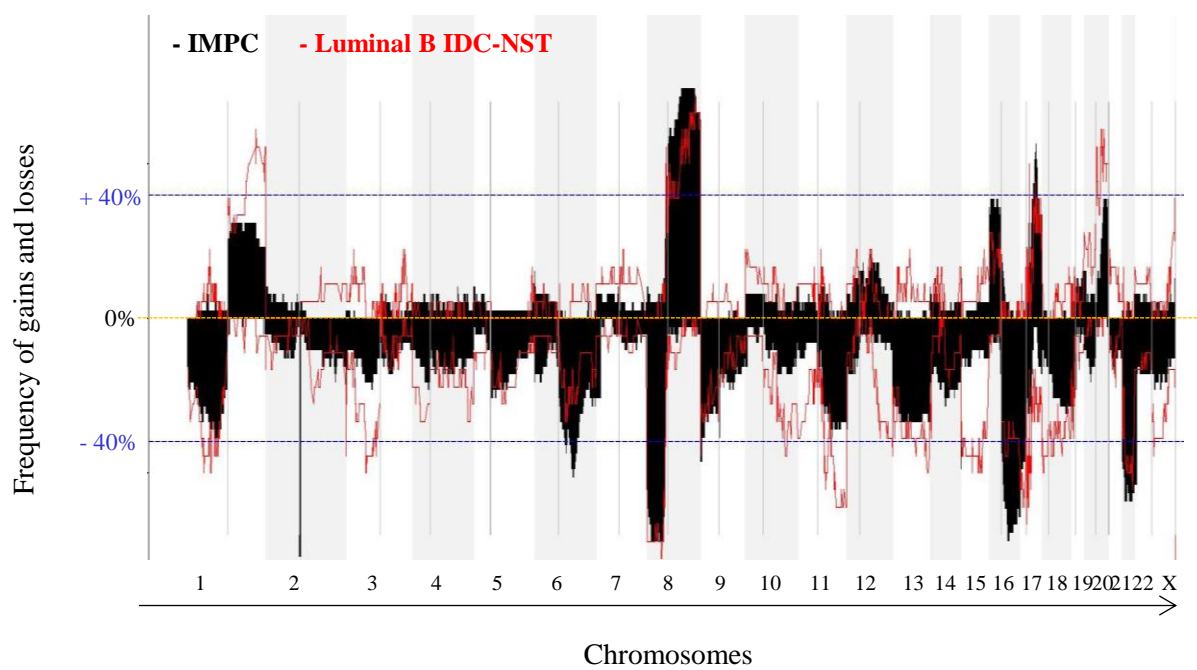

**Supplementary Figure 3**

Supplement: Additional file 12: Figure S3 — Clinical, pathological characteristics, and treatments of patients and tumours in the two genomic subgroups of invasive micropapillary carcinoma. [file bcr3653-S12.pdf]
