# Supplementary material for: Polarity gene alterations in pure invasive micropapillary carcinomas of the breast
Source: Breast Cancer Res. 2014 May 8;16(3):R46. doi: 10.1186/bcr3653 (PMC4095699; doi:10.1186/bcr3653)
Supplement: Additional file 7: Table S2 — Frequencies of common and specific gains and losses in Sawtooth/8/16 and Firestorm/Amplifier invasive micropapillary carcinoma subsets. [file bcr3653-S7.pdf]

Supplementary table 2: Frequencies of common and specific gains and losses in Sawtooth/8/16 and Firestorm/Amplifier IMPC subsets.

	Pos SNP Start	Pos SNP End	Chr	Cytoband	Sawtooth/8/16 (%)	Firestorm/Amplifier (%)	Genes of interest
**Common regions**							
Gains							
	36872094	146293414	8	p11.23-q24.3	83	53	
Losses							
	315843	35282003	8	p23.3-p12	62	66	
	46534977	90163275	16	q11.2-q24.3	74	40	
**Specific regions**							
Gains							
	552621	35205717	16	p13.3-p11.1	71	8	
	25899668	28036350	17	q11.2	0	44	
	35306175	38327133	17	q12-q21.1	0	59	
	47177747	81049725	17	q21.32-q25.3	18	62	
	46889933	62912463	20	q13.13-q13.33	18	51	
Losses							
	34811050	114150717	1	p34.3-p13.2	8	49	
	70106360	170918031	6	q13-q27	50*	23*	
	2310130	22235650	17	p13.3-p11.1	15	74	
	32541260	35320846	17	q12	13	43	
	41982253	46948218	17	q21.31-31.32	13	41	
	34734484	77828600	18	q12.2-q23	11	42	
**Amplifications**							
	94690198	132251807	8	q22.1-q24.22	2	35	*RAD54B, CCNE2, TP53INP1, RAD21,* [illegible]
	37251066	38303547	17	q12-q21.1	0	37	*STARD3, ERBB2, GRB7*
	48579452	49994258	17	q21.33	5	20	
	57486793	62354992	17	q22-q23.3	6	46	*PPM1D, BCAS3, TBX2, TBX4, TLK1,* [illegible]
	48483623	49500894	20	q13.13	0	23	*BCAS4*
	50796522	53259964	20	q13.2	0	30	*ZNF217, BCAS1*

Legends: Recurrent gains, losses or amplifications regions after exclusion of genomic variant according to the DGV database, observed in more than 40% of cases. Pos SNP Start/ pos SNP End: position of the SNP that represent the boundaries of gains, losses or amplifications. Genomic positions are provided according to human genome 19 references in bp; Chr: chromosome; IMPC: invasive micropapillary carcinoma; *: losses associated with loss of heterozygosity (LOH).
