# Supplementary material for: Polarity gene alterations in pure invasive micropapillary carcinomas of the breast
Source: Breast Cancer Res. 2014 May 8;16(3):R46. doi: 10.1186/bcr3653 (PMC4095699; doi:10.1186/bcr3653)
Supplement: Additional file 8: Table S3 — Frequencies of common and specific regions of gains and losses in Firestorm/Amplifier invasive micropapillary carcinoma subgroup and invasive ductal carcinoma of no special type. [file bcr3653-S8.pdf]

Supplementary Table 3: Frequencies of common and specific regions of gains and losses in Firestorm/Amplifier IMPC subgroup and IDC-NST.

Pos SNP Start	Pos SNP End	Chr	Cytoband	Firestorm/Amplifier (%)	IDC-NST (%)
**Common regions**					
Gains					
154625632	249198692	1	q21.3-q44	41	43
36948617	38618768	8	p11.23-p11.22	45	42
43614559	62912463	20	q13.12-q13.33	48	45
Losses					
26214603	113346108	1	p36.11-p13.2	50	40
113565	35323410	8	p23.3-p12	65*	60*
80033803	134944770	11	q14.1-q25	45*	45*
39949125	115106996	13	q13.3-q34	41*	40*
46534977	90163275	16	q11.2-q24.3	41*	54*
16055171	51219006	22	q11.1-q13.33	61*	40*
**Specific regions**					
Gains					
25311244	28026491	17	q11.1-q11.2	43	10
35306175	38256973	17	q12-q21.1	60	18
46954793	81049725	17	q21.32-q25.3	62	15
33435161	38150561	20	q11.22-q12	13	43
Losses					
6689	22235650	17	p13.3-p11.1	78*	41*

Legends: Recurrent gains, losses or amplifications regions after exclusion of genomic variant according to the DGV database, observed in more than 40% of cases. Pos SNP Start/ pos SNP End: position of the SNP that represent the boundaries of gains, losses or amplifications. Genomic positions are provided according to human genome 19 references in bp ; Chr: chromosome ; IMPC: invasive micropapillary carcinoma ; IDC-NST: invasive carcinomas of no special type; *: losses associated with loss of heterozygosity (LOH).
