# Supplementary material for: Polarity gene alterations in pure invasive micropapillary carcinomas of the breast
Source: Breast Cancer Res. 2014 May 8;16(3):R46. doi: 10.1186/bcr3653 (PMC4095699; doi:10.1186/bcr3653)
Supplement: Additional file 9: Table S4 — Frequencies of common and specific regions of gains and losses in Sawtooth/8/16 invasive micropapillary carcinoma subgroup and invasive ductal carcinoma of no special type. [file bcr3653-S9.pdf]

Supplementary Table 4: Frequencies of common and specific regions of gains and losses in Sawtooth/8/16 IMPC subgroup and IDC-NST.

Pos SNP Start	Pos SNP End	Chr	Cytoband	Sawtooth/8/16 (%)	IDC-NST (%)
**Common regions**					
Losses					
113565	35436036	8	p23.3-p12	63*	61*
23921202	51219006	22	q11.23-q13.33	49*	43*
**Specific regions**					
Gains					
197811282	249198692	1	q31.3-q44	6	48
36694951	146292734	8	p11.23-q24.3	80	45
86671	35205717	16	p13.3-p11.1	68	25
33435161	62648208	20	q11.22-q13.33	7	44
Losses					
45477675	99170631	1	p34.1-p21.3	11	42
70456678	170918031	6	q13-q27	50*	16*
80033803	134944770	11	q14.1-q25	22*	47*
46534977	90163275	16	q11.2-q24.3	79*	54*
531729	20030960	17	p13.3-p11.2	13*	43*

Legends: Recurrent gains, losses or amplifications regions after exclusion of genomic variant according to the DGV database, observed in more than 40% of cases. Pos SNP Start/ pos SNP End: position of the SNP that represent the boundaries of gains, losses or amplifications. Genomic positions are provided according to human genome 19 references in bp; Chr: chromosome; IMPC: invasive micropapillary carcinoma ; IDC-NST: invasive carcinomas of no special type; *: losses associated with loss of heterozygosity (LOH).
