# Supplementary material for: Polarity gene alterations in pure invasive micropapillary carcinomas of the breast
Source: Breast Cancer Res. 2014 May 8;16(3):R46. doi: 10.1186/bcr3653 (PMC4095699; doi:10.1186/bcr3653)
Supplement: Additional file 10: Table S5 — Comparison of frequency plots of invasive micropapillary carcinoma and luminal B invasive ductal carcinoma of no special type. Frequency plots of gains and losses are displayed from chromosome 1pter on the left to chromosome Xq on the right. Alternating grey and white bands indicate chromosome boundaries. Dashed blue line represent 40% frequencies, − for losses and + for gains, respectively. IMPC, Invasive micropapillary carcinomas; Luminal B IDC-NST, Luminal B invasive ductal carcinoma of no special type. [file bcr3653-S10.pdf]

Supplementary Table 5: Clinical, pathological characteristics and treatments of patients and tumors of the two genomic sub-groups of IMPC.

	**Sawtooth / 8/16 / [n (%)]**	**Firestorm/Amplifier/ [n (%)]**	***p-val***
Number of patients	16	19	
Median FU, yrs [range]	5.9 [0.6 - 10.2]	7.5 [1.1 - 10.7]	
Median age, yrs [range]	61 [39 - 84]	62 [34 - 83]	
< 50	1 (6)	6 (32)	ns
> 50	15 (94)	13 (68)	
Tumor size, cm			
< 2	12 (75)	12 (63)	ns
2 to 5	3 (19)	6 (32)	ns
> 5	1 (6)	1 (5)	ns
Grade			
I	2 (13)	0 (0)	ns
II	7 (44)	11 (58)	ns
III	7 (44)	7 (37)	ns
Not specified	0 (0)	1 (5)	
Nodal status			
Positive	9 (56)	11 (58)	ns
Negative	6 (38)	7 (37)	
Not specified	1 (6)	1 (5)	
Lyhovascular invasion			
Positive	14 (88)	15 (79)	ns
Negative	2 (13)	3 (16)	
Not specified	0 (0)	1 (5)	
Conservative surgery	11 (69)	10 (53)	ns
Adjuvant therapies			
Anti-HER2 therapy	1 (100)	4 (44)	ns
Chemotherapy	6 (38)	12 (63)	ns
Hormone treatment	15 (94)	16 (84)	ns
ER			
Positive	16 (100)	19 (100)	ns
Negative	0 (0)	0 (0)	
ERBB2			
Positive	1 (6)	9 (47)	7.3×10^{-3}
Negative	13 (81)	9 (47)	
Not specified	2 (13)	1 (5)	
