# Supplementary material for: Polarity gene alterations in pure invasive micropapillary carcinomas of the breast
Source: Breast Cancer Res. 2014 May 8;16(3):R46. doi: 10.1186/bcr3653 (PMC4095699; doi:10.1186/bcr3653)
Supplement: Additional file 11: Table S7 — Frequencies of common and specific regions of gains and losses in invasive micropapillary carcinoma and luminal B invasive ductal carcinoma of no special type. [file bcr3653-S11.pdf]

Supplementary Table 7: Frequencies of common and specific regions of gains and losses in IMPC and luminal B IDC-NST.

Pos SNP Start	Pos SNP End	Chr	Cytoband	IMPC	lum B IDC-NST
**Common regions**					
Gains					
47043376	146263538	8	q11.21-q24.3	66	51
49959379	64819791	17	q22-q24.2	48	33
Losses					
103565	33777560	8	p23.3-p12	63	66
6689	18837022	17	p13.3-p11.2	46	53
25196483	29511625	17	q11.2	24	53
23664977	49565872	22	q11.23-q13.32	55	47
45092478	88690776	16	q11.2-q24.2	55	37
**Specific regions**					
Gains					
224028565	238251069	1	q42.12-q43	25	56
32596025	62341267	20	q11.22-q13.33	25	50
Losses					
47042807	73900069	3	p21.31-p13	19	43
106624649	109132959	6	q21	50	19
21506625	85593332	15	q11.2-q25.2	11	44
400368	13601533	18	p23.3_p22	12	46
26429175	28443413	18	q12	16	44

Legends: Recurrent gains and losses regions after exclusion of genomic variant according to the DGV database, observed in more than 40% of cases. Pos SNP Start/ pos SNP End: position of the SNP that represent the boundaries of gains, losses or amplifications. Genomic positions are provided according to human genome 19 references in bp; Chr: chromosome; IMPC: invasive micropapillary carcinoma; IDC-NST: invasive carcinomas of no special type; Luminal B IDC-NST were selected according to Von Minckwitz method [1] *: losses associated with loss of heterozygosity (LOH).

1. **von Minckwitz G, Untch M, Blohmer JU, Costa SD, Eidtmann H, Fasching PA, Gerber B, Eiermann W, Hilfrich J, Huober J *et al*: Definition and impact of pathologic complete response on prognosis after neoadjuvant chemotherapy in various intrinsic breast cancer subtypes. *J Clin Oncol* 2012, 30(15):1796-1804.**
